# Post-Mortem Examination of an Apoplectic Patient

**Published:** 1826-03

**Authors:** R. Wade

**Affiliations:** Member of the Royal College of Surgeons, and Apothecary to the Westminster General Dispensary.


ART. II.—*Post-mortem Examination of an Apoplectic Patient.* By R. WADE, Member of the Royal College of Surgeons, and Apothecary to the Westminster General Dispensary.

POST-MORTEM examinations of persons who die apoplectic, too often add but little to our pathological knowledge; the appearances on dissection being frequently insufficient to account for the phenomena of the disease. When, however, the result of such examinations is satisfactory, a statement of the case cannot fail to be interesting; and as, in the following instance, not only more extensive injury in the brain than is generally found existed, but also an unusual state of disease in the plexus choroides, I am induced to request the favour of its insertion in the Medical and Physical Journal.

William Richardson, forty-two years of age, a strong muscular man, of a plethoric habit, by occupation a leather-dresser, and enjoying generally a good state of health, never having had any serious illness, (with the exception of a bowel-complaint twelve months ago,) until the present attack. On his return from work on the night of the 3d of January, he complained of being very unwell. On being questioned as to the nature of his illness, he said that he had a slight degree of pain in the head, and desired to have some warm gruel. His wife says, she had no reason to suppose, from her husband's appearance, that he had been drinking to any excess, either on this or any preceding day: on the contrary, she had always considered him a man of temperate habits. After having taken the gruel, he went to bed, and about one o'clock in the morning awoke his wife: he was then breathing with great difficulty, appeared very restless, and got out of bed, but immediately fell. Assistance was procured, and he was replaced in his bed, in a state of insensibility.

On my first visiting the patient, at ten o'clock in the morning (Jan. 4th), I found him apoplectic, with stertorous breathing, countenance extremely sunk, and of a pale blue colour; the pulse at the wrist was slow and oppressed; the pupils dilated; the whole venous system appeared overcharged. On opening a vein in the arm, the blood gushed out as on the division of an artery, and thirty ounces were very quickly taken. The bleeding diminished the force, and increased the frequency, of the pulse; but not the slightest degree of relief was obtained by it. He was then ordered to be cupped to sixteen ounces at the nape of the neck, and ten grains of calomel were ordered; only a part of which, however, could be administered. An enema was also prescribed.

I saw him again at three o'clock, and found that no relief had been obtained: indeed, he was evidently dying; and expired at eight o'clock in the evening. I examined the body early next morning, and the following were the appearances which presented themselves:—

On removing the dura mater, which adhered very firmly to the cranium, the whole venous system on the surface of the brain appeared excessively distended; the tunica arachnoidea, over the anterior cerebral lobes, was of a dirty yellowish colour, from lymph which had been effused under it. I cannot convey a better idea of the appearance which the brain presented through the tunica arachnoidea and pia mater, than by comparing it to a damson-pudding: this appearance was produced by the congested state of the vessels of the cerebrum, and by the dark blood effused between the convolutions. On removing the tunica arachnoidea and pia mater, which were firmly agglutinated together in every part, and were thicker than the dura mater, the under surface of the pia mater strongly resembled the mucous membrane of the intestines in a high state of inflammation; the pia mater itself being of a dark crimson colour, whilst the veins, which were in every part distended to the size of a common quill, adhering firmly to the membrane, resembled in some parts the injected valvulæ conniventes, and in others the rugæ of an inflamed stomach. The whole internal surface of the pia mater was covered with a thin layer of venous blood, coated in many places with lymph. The effusion of lymph appeared to have taken place chiefly over the coats of the veins.—On opening the right ventricle, the corpus striatum was observed to be of a bluish-black colour: in its centre was a small portion of coagulated blood, double the size of a pea; the part round the coagulum presented the appearance of kidney. In the plexus choroides was a small tumor, the size of a large pea, which, on being cut, was found to consist chiefly of an earthy substance, and appeared like a small gland, thickly interspersed with a calcareous matter. The thalamus nervi optici was healthy.—In the left ventricle, the pia mater, particularly in the anterior cornu, had a speckled appearance, from ecchymosis. On dividing the corpus striatum on this side, a coagulum of blood, larger than that in the right ventricle, was found in its anterior portion; and the parts around it had the same kidney-like aspect as the other. The internal part of the thalamus had a mottled appearance, from little spots of ecchymosis. The plexus choroides on this side contained a tumor, precisely similar to that in the right ventricle; but the plexus itself was of a lighter colour.—No serum was contained in any of the ventricles; the middle one was quite free from disease. The velum interpositum was highly injected. The pia mater, on the base of the cerebrum, was in the same state as on its surface; as also that portion investing the cerebellum. All the sinuses were distended. Under the anterior part of the right lobe of the cerebellum, a small quantity of coagulated blood was observed. A careful examination was made of the arteries at the base of the brain: no ossification, however, was found.

The heart was rather larger than usual; the lungs healthy; about half a pint of bloody serum in the right pleura; the viscera of the abdomen sound.

